# Cytochrome c oxidase deficiency accelerates mitochondrial apoptosis by activating ceramide synthase 6

**DOI:** 10.1038/cddis.2015.62

**Published:** 2015-03-12

**Authors:** S Schüll, S D Günther, S Brodesser, J M Seeger, B Tosetti, K Wiegmann, C Pongratz, F Diaz, A Witt, M Andree, K Brinkmann, M Krönke, R J Wiesner, H Kashkar

**Affiliations:** 1 Center for Molecular Medicine Cologne (CMMC) and Institute for Medical Microbiology, Immunology and Hygiene (IMMIH), University of Cologne, Cologne, Germany; 2 Cologne Excellence Cluster on Cellular Stress Responses in Aging-Associated Diseases (CECAD) Research Center, University of Cologne, Cologne, Germany; 3 Department of Neurology, University of Miami, Miami, FL, USA; 4 Institute for Vegetative Physiology, University of Cologne, Cologne, Germany

## Abstract

Although numerous pathogenic changes within the mitochondrial respiratory chain (RC) have been associated with an elevated occurrence of apoptosis within the affected tissues, the mechanistic insight into how mitochondrial dysfunction initiates apoptotic cell death is still unknown. In this study, we show that the specific alteration of the cytochrome c oxidase (COX), representing a common defect found in mitochondrial diseases, facilitates mitochondrial apoptosis in response to oxidative stress. Our data identified an increased ceramide synthase 6 (CerS6) activity as an important pro-apoptotic response to COX dysfunction induced either by chemical or genetic approaches. The elevated CerS6 activity resulted in accumulation of the pro-apoptotic C_16 : 0_ ceramide, which facilitates the mitochondrial apoptosis in response to oxidative stress. Accordingly, inhibition of CerS6 or its specific knockdown diminished the increased susceptibility of COX-deficient cells to oxidative stress. Our results provide new insights into how mitochondrial RC dysfunction mechanistically interferes with the apoptotic machinery. On the basis of its pivotal role in regulating cell death upon COX dysfunction, CerS6 might potentially represent a novel target for therapeutic intervention in mitochondrial diseases caused by COX dysfunction.

The mitochondrial oxidative phosphorylation (OXPHOS) machinery is composed of four multisubunit respiratory complexes (complex I–IV) creating an electrochemical gradient through the coupled transfer of electrons to oxygen and the transport of protons from the matrix across the inner mitochondrial membrane (IMM) into the intermembrane space (IMS), used by the ATP-synthase to produce ATP.^1^ The components of the OXPHOS machinery are encoded by nuclear DNA (nDNA) as well as by mitochondrial DNA (mtDNA). Mutations in either nuclear or mitochondrial genes result in mitochondrial dysfunction and precipitate versatile severe degenerative diseases, premature aging phenotypes and mortality.^2, 3^ It is increasingly evident that defects in OXPHOS results in degenerative states based on the accelerated apoptotic death of the damaged cells. However, while numerous pathogenic changes within OXPHOS have been associated with an elevated occurrence of apoptosis, the causal role of mitochondrial dysfunction in the initiation of the apoptotic program and the underlying molecular mechanism linking OXPHOS dysfunction to the cellular apoptotic machinery are incompletely understood.^3^

Apoptosis is a mode of cell death that is used by multicellular organisms to dispose irreparably damaged cells and is executed by a family of proteases known as caspases.^4^ Caspase activity can be either initiated extrinsically or intrinsically. Extrinsic apoptosis is triggered by binding of extracellular ligands to tumor necrosis factor receptor family members and results in the activation of the initiator caspase, caspase-8.^5^ Overwhelming cellular damage may alternatively initiate the death of the cell autonomously by involving mitochondria (intrinsic apoptotic pathway). Accordingly, intrinsic stress cues promote mitochondrial outer membrane permeabilization (MOMP), a process which is tightly regulated by members of the Bcl-2 protein family^6^ and results in the release of pro-apoptotic factors from IMS including cytochrome c. Cytosolic cytochrome c subsequently initiates the activation of the initiator caspase, caspase-9.^7^ Once active, these initiator caspases cleave and activate the zymogens of executioner caspases such as caspase-3, which in turn are responsible for the majority of proteolytic events that ultimately result in the apoptotic destruction of the cell.^4^

Cytochrome c oxidase (COX or complex IV) is a 200-kDa multicomponent enzyme located in the IMM and represents the terminal complex of the electron transport chain. COX is composed of 13 subunits encoded by both the mitochondrial (subunits 1, 2 and 3, which form the catalytic core of the enzyme) and the nuclear genomes.^8^ Naturally occurring COX dysfunctions are predominantly caused by somatic or inherited mutations in the mtDNA and nDNA. Here we show that COX deficiency, which represents a common defect found in mitochondrial diseases, increases the mitochondrial apoptotic response to oxidative stress. Our data identify elevated levels of ceramide with an acyl chain of C_16 : 0_ to be responsible for the increased susceptibility of COX-deficient cells to oxidative stress. Specifically, COX dysfunction enhances ceramide synthase 6 (CerS6) transcription and activity, which in turn results in C_16 : 0_ ceramide accumulation, cytochrome c release and accelerated apoptosis in response to oxidative stress. Our results provide new insights into how mitochondrial dysfunction mechanistically interferes with the apoptotic machinery and how it induces the apoptotic demise of damaged cells in an affected degenerating tissue.

## Results

### Inhibition of COX increases cellular susceptibility to H_2_O_2_

**Figure 1:**
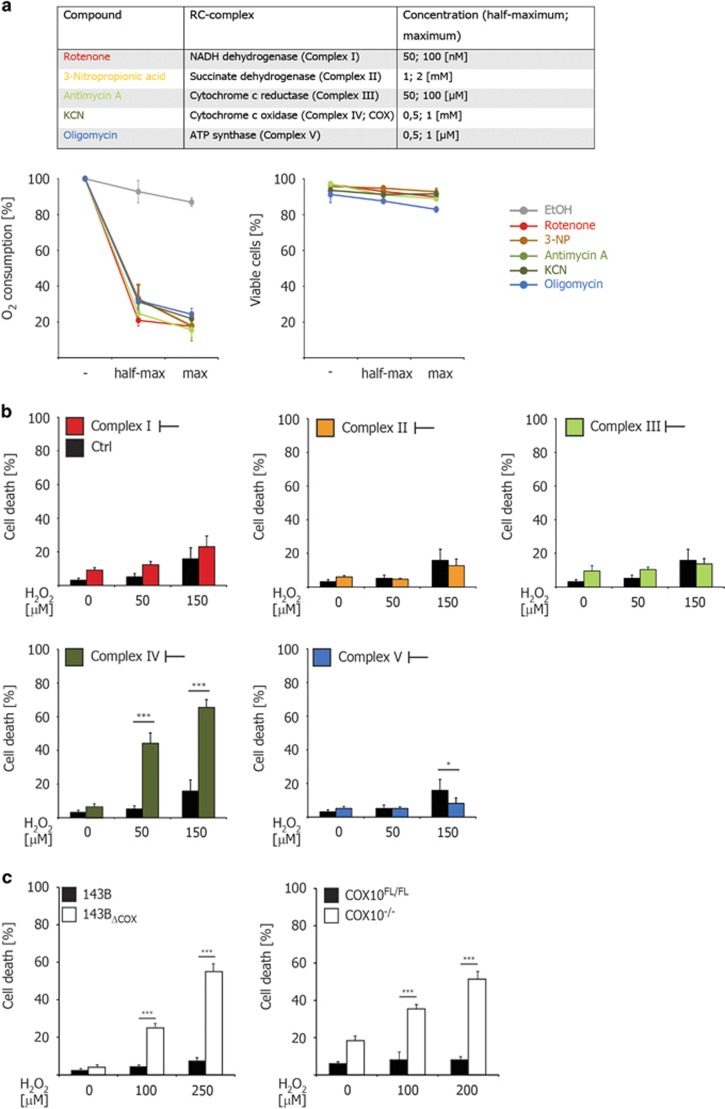
COX deficiency enhances susceptibility to H_2_O_2_-induced cell death. (**a**) List of utilized chemical inhibitors of the mitochondrial RC. Inhibition of individual RC complexes in HeLa cells was quantified by a reduction in oxygen consumption (left panel). Cytotoxicity was quantified by trypan-blue exclusion after 48 h (right panel). (**b**) Analysis of cell death by trypan-blue exclusion in HeLa cells treated for 24 h with individual RC complex inhibitors as indicated and subjected to H_2_O_2_ for further 24 h. (**c**) Analysis of cell death by trypan-blue exclusion in 143B_ΔCOX_ cybrid cells and COX10^−/−^ fibroblasts subjected to H_2_O_2_ for 20 h. The error bars in panel **a** and **c** represent the mean±S.D. (*n*=3), the error bars in panel **b** represent the mean±S.D. (*n*=6), two-tailed unpaired *t*-test. *P <0.05, ***P<0.001

We first investigated whether the alteration of mitochondrial respiration in general or the specific inhibition of one of the mitochondrial respiratory chain (RC) complexes directly impacts on cellular viability. Different RC complexes were chemically inhibited in HeLa cells and oxygen consumption was monitored to examine the efficacy of the inhibition (Figure 1a). Although efficiently reducing oxygen consumption, none of the tested chemical inhibitors of RC exhibited significant cytotoxic activity within 48 h, suggesting that the inhibition of RC *per se* does not directly activate the cellular death program in highly glycolytic HeLa cells.^9^ The elevated occurrence of apoptosis within a tissue upon mitochondrial dysfunction may additionally arise from an increased susceptibility of cells toward environmental cues such as oxidative stress. Indeed, physiologic levels of reactive oxygen species (ROS) are able to promote oxidative stress and cause lethality when cellular integrity or fitness is altered, for example, upon aging.^10, 11^ Therefore, we examined whether RC inhibition impacts on cellular sensitivity to ROS. Accordingly, HeLa cells were first exposed to different inhibitors of RC complexes (24 h) and subsequently treated with H_2_O_2_ (24 h). In striking contrast to complex I, II, III and V, the inhibition of the respiratory complex IV (COX) by KCN markedly increased the susceptibility of cells toward H_2_O_2_ treatment (Figure 1b and Supplementary Figure 1).

In order to substantiate our observations, two cell lines with genetic alteration of COX, the human osteosarcoma cybrid cell line 143B_ΔCOX_ (mtDNA mutation leading to inactivation of subunit 1 of COX, COXI)^12^ and the murine fibroblasts COX10^−/−^ (conditional inactivation of nDNA-encoded subunit 10 of COX, COX10)^13^ were employed to examine the role of COX dysfunction in susceptibility toward oxidative stress (Figure 1c). Similar to KCN-treated HeLa cells, COX dysfunction in both 143B_ΔCOX_ and COX10^−/−^ cells resulted in an increased susceptibility to H_2_O_2_ treatment.

### COX dysfunction accelerates mitochondrial apoptosis in response to oxidative stress

**Figure 2:**
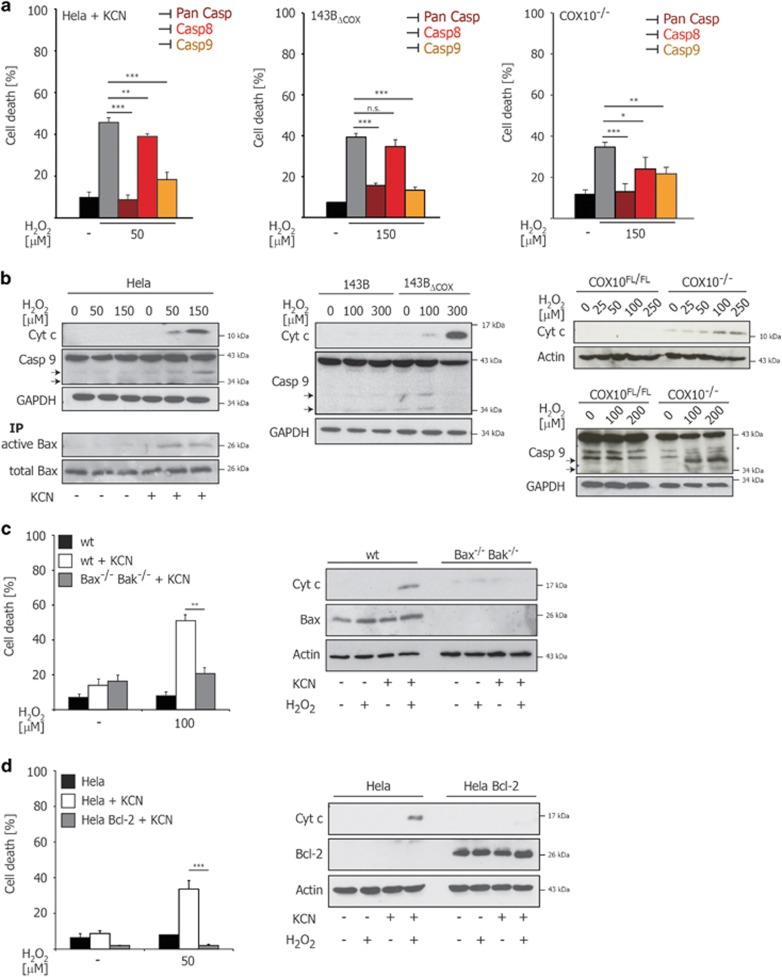
H_2_O_2_ induces mitochondrial apoptosis in COX-deficient cells. (**a**) HeLa cells pretreated with KCN (1 mM) for 24 h, 143B_ΔCOX_ cybrid cells and COX10^−/−^ fibroblasts were subjected to H_2_O_2_ in the absence (gray column) or presence of pan-caspase inhibitor zVAD-fmk (20 *μ*M), caspase-8 inhibitor zIETD-fmk (2 *μ*M) or caspase-9 inhibitor zLEHD-fmk (2 *μ*M). Cell death was measured by trypan-blue exclusion after 20 h. (**b**) Cytchrome c and caspase-9 were detected in the cytosol of cells by WB after treatment with H_2_O_2_ for 6 h (HeLa), 12 h (143B_ΔCOX_ cybrid cells) or 16 h (COX10^−/−^), respectively. The arrowheads indicate caspase-9 cleavage bands p35 and p37. Activated Bax was immunoprecipitated by 6A7 anti-Bax antibody in total lysates of HeLa cells and detected by WB after 4 h. (**c**) Wild-type (wt) and Bax/Bak double-knockout MEFs (Bax^−/−^Bak^−/−^) were pretreated with KCN (200 *μ*M) for 24 h or left untreated. Cell death upon treatment with H_2_O_2_ was measured after 18 h by trypan-blue exclusion. The release of cytochrome c was detected by WB after 6 h. (**d**) HeLa and HeLa Bcl-2 overexpressing cells were pretreated with KCN (1 mM) for 24 h or left untreated. Cell death upon treatment with H_2_O_2_ was measured after 18 h by trypan-blue exclusion and release of cytochrome c was detected by WB after 6 h. The error bars in panel **a** represent the mean±S.D. (*n*=4), the error bars in panel **c** and **d** represent the mean±S.D. (*n*=3), two-tailed unpaired *t*-test. *P<0.05, **P <0.01, ***P<0.001

To test whether the increased susceptibility to oxidative stress upon COX inhibition was due to enhanced apoptosis, we examined cell death in the presence of caspase inhibitors (Figure 2a). Remarkably, the pan-caspase inhibitor zVAD completely abolished the susceptibility to H_2_O_2_ in KCN-treated HeLa, 143B_ΔCOX_ and COX10^−/−^ cells. Furthermore, the susceptibility of COX-deficient cells was uniformly diminished when the initiator caspase-9 was inhibited, but not caspase-8, indicating the involvement of the mitochondrial apoptotic machinery. Indeed, KCN treatment in HeLa cells facilitated Bax activation, the release of cytochrome c and caspase-9 activation upon H_2_O_2_ treatment (Figure 2b, left panel). Similarly, accelerated cytochrome c release and caspase-9 activation were observed in 143B_ΔCOX_ and COX10^−/−^ cells after H_2_O_2_ treatment (Figure 2b, middle and right panels). The mitochondrial apoptotic pathway converges on the two pro-apoptotic Bcl-2 proteins Bax and Bak, either of which is sufficient to drive MOMP and induce cytochrome c release from mitochondria.^14^ To further investigate the involvement of the mitochondrial apoptotic pathway, Bax- and Bak-deficient murine embryonic fibroblasts (Bax^−/−^Bak^−/−^ MEFs)^15^ and HeLa cells stably overexpressing the anti-apoptotic Bcl-2 protein (HeLa Bcl-2)^16^ were analyzed. In both Bax^−/−^Bak^−/−^ MEFs and HeLa Bcl-2 cell line, COX inhibition by KCN treatment did neither enhance cell death nor promote cytochrome c release in response to H_2_O_2_ (Figures 2c and d). These data conclusively demonstrate that COX inhibition accelerates mitochondrial apoptotic response to oxidative stress.

### COX dysfunction increases cellular ceramide levels

In order to examine whether the increased susceptibility to oxidative stress is due to elevated basal levels of ROS upon COX dysfunction, we analyzed the amounts of mitochondrial superoxide in KCN-treated HeLa, 143B_ΔCOX_ and COX10^−/−^ cell lines (Supplementary Figure 2A). Consistent with previous observations,^17^ no alterations in cellular ROS levels were observed in COX-deficient cells. Furthermore, the expression levels of the Bcl-2 protein family members, as the major regulators of the mitochondrial apoptotic pathway, were investigated in cells lacking functional COX (Supplementary Figure 2B). These analyses showed that COX dysfunction did not provoke any alteration in the expression pattern of pro- or anti-apoptotic Bcl-2 proteins that would explain the increased susceptibility of these cells toward oxidative stress.

**Figure 3:**
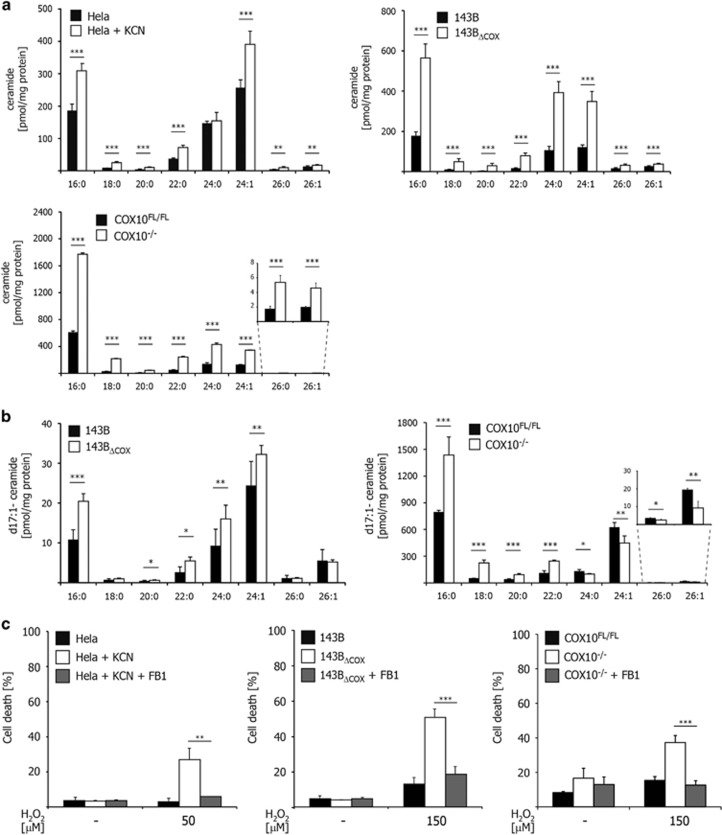
COX deficiency leads to the accumulation of pro-apoptotic ceramides. (**a**) Total ceramide content of COX-deficient cells and their respective controls was analyzed by mass spectrometry in total cell homogenates. HeLa cells were pretreated with KCN (1 mM) for 48 h or left untreated. (**b**) Total ceramide synthase activity in COX-deficient cells and their respective controls was measured by the incorporation of d17 : 0 dihydrosphingosine into the sphingoid backbone of ceramides (d17 : 1 ceramides). Incorporation of d17 : 0 dihydrosphingosine (2 *μ*M) into ceramides was assessed after 4 h by mass spectrometry of total cell homogenates. (**c**) COX-deficient cells were incubated in the presence of the ceramide synthase inhibitor FB1 (HeLa 20 *μ*M, 143B_ΔCOX_ 30 *μ*M and COX10^−/−^ 50 *μ*M) and HeLa cells were incubated additionally with KCN (1 mM) for 24 h. Cell death upon treatment with H_2_O_2_ was measured after 20 h by trypan-blue exclusion. Error bars in panel **a** represent the mean±S.D. of two analytical replicates of three biological replicates (HeLa cells and 143B cybrids) and mean±S.D. of two analytical replicates of two biological replicates (COX10), respectively. Error bars in panel **b** represent mean±S.D. of two analytical replicates of four biological (143B cybrids) and two biological (COX10) replicates, respectively. Error bars in panel **c** represent mean±S.D. (*n*=3), two-tailed unpaired *t*-test. *P<0.05, **P<0.01, ***P<0.001

Changes in lipid composition of mitochondrial membranes are intimately associated with MOMP and substantially impact on cellular apoptotic response, in particular, upon oxidative stress.^18^ Specifically, emerging evidence indicated that sphingolipid metabolism controls Bax/Bak-mediated cytochrome c release and mitochondrial apoptosis.^19, 20, 21, 22, 23^ Therefore, we conducted an extensive mass spectrometry analysis of cellular sphingolipids in KCN-treated HeLa, 143B_ΔCOX_ and COX10^−/−^ cells to monitor the possible modifications of cellular sphingolipid contents upon COX dysfunction (Figure 3a and Supplementary Figure 3A). The data obtained showed that short- and long-chain ceramide species accumulate when COX was inhibited (Figure 3a).

Ceramides represent important sphingolipid metabolites acting as precursors for the synthesis of sphingomyelins and cerebrosides similar to glucosylceramide.^24^ Ceramides are generated via *N*-acylation of dihydrosphingosine by ceramide synthases, forming dihydroceramide, which is subsequently dehydrogenated to ceramides by dihydroceramide desaturase (*de novo* pathway). Alternatively, ceramides can be regenerated via sphingomyelin hydrolysis by sphingomyelinases (SMase) at the plasma membrane or via degradation of preformed sphingolipids to sphingosine, which in turn can be used by ceramide synthases (salvage pathway).^25^ Our data showed, however, that ceramide accumulation upon COX inhibition was mainly mediated via the activity of ceramide synthase. Accordingly, no reduction of cellular sphingomyelin content or upregulation of sphingomyelinase activity could be detected when COX was inhibited (Supplementary Figures 3A and B). Furthermore, these analyses even revealed elevated sphingomyelin levels in COX-deficient cells as a consequence of the increased cellular ceramide content.

In order to examine the role of ceramide synthase activity in this process, we adapted a previously described protocol to analyze ceramide synthesis in viable cells by using d17 : 0 dihydrosphingosine and mass spectrometry.^26^ C_17_-sphingosine is one carbon shorter than naturally occurring C_18_-sphingosine, allowing its metabolism to ceramide to be followed in intact cells by mass spectrometry. The tracing of the incorporation of exogenously added d17 : 0 dihydrosphingosine indicated a significant increase of ceramide synthase activity in 143B_ΔCOX_ and COX10^−/−^ cell lines compared with the respective control cell lines (Figure 3b). Together, these data show that COX deficiency increases cellular ceramide levels by enhancing ceramide synthase activity.

Ceramides contribute to MOMP either lone by forming ceramide channels in mitochondria outer membrane or by involving pro-apoptotic Bax/Bak. Strikingly, ceramide channel formation is specific to mitochondrial membranes in that no channel formation was observed, for example, in the plasma membranes of erythrocytes even at concentrations 20 times higher than those required for channel formation in mitochondrial outer membranes.^27^ The involvement of ceramide synthesis in COX deficiency-induced susceptibility to oxidative stress was further analyzed by mycotoxin fumonisin B1 (FB1), a competitive inhibitor of sphingosine and dihydrosphingosine for binding to ceramide synthases.^28^ FB1 almost completely restored the increased susceptibility of COX-deficient cells toward oxidative stress (Figure 3c and Supplementary Figure 3C) underscoring the role of ceramide synthesis in this process.

### CerS6 is responsible for the increased susceptibility to oxidative stress upon COX dysfunction

**Figure 4:**
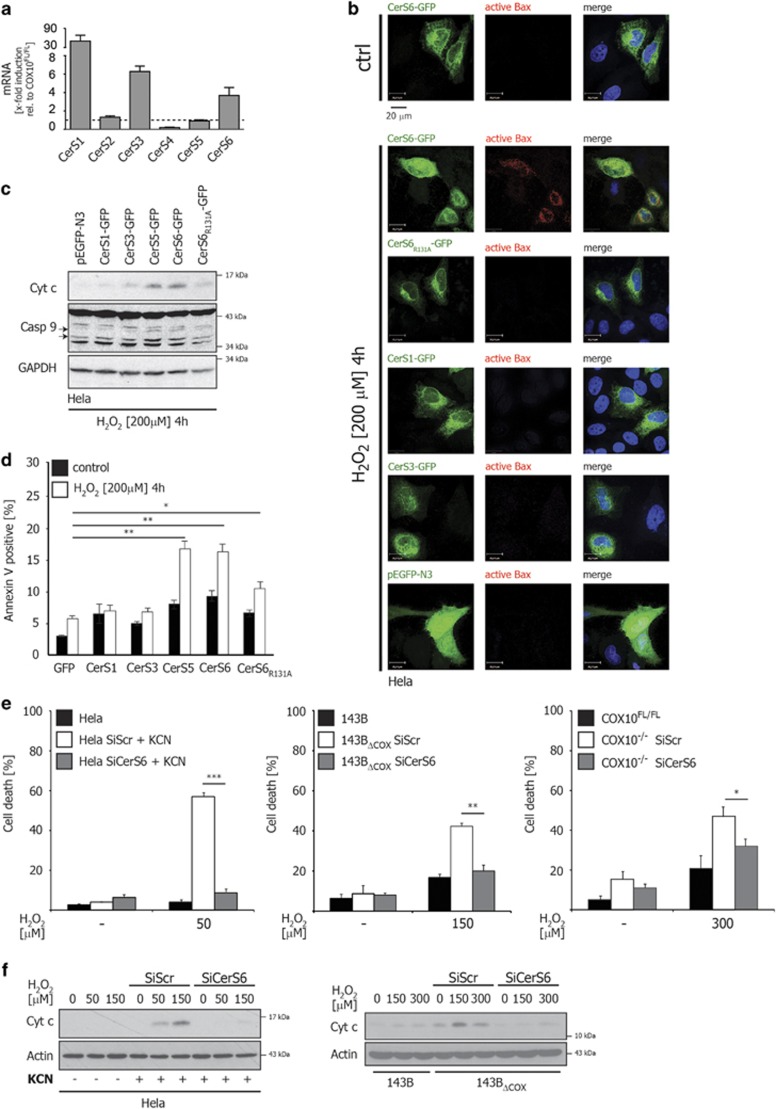
CerS6 is responsible for COX deficiency-induced apoptosis in response to oxidative stress. (**a**) Total RNA was isolated from COX10^fl/fl^ and COX10^−/−^ fibroblasts and transcribed into cDNA using Oligo(dT) primers. qPCR was performed using primers specific for each of the six mammalian ceramide synthases and normalized to GAPDH. Represented is mean±S.D. from three experimental replicates relative to COX10^fl/fl^. (**b**) HeLa cells were transiently transfected with human CerS6-GFP and left untreated (upper row). Transiently transfected HeLa cells (transfection efficiency was up to 50%) with human CerS6-GFP, CerS6_R131A_-GFP, CerS1-GFP, CerS3-GFP or empty vector (pEGFP-N3) were treated with H_2_O_2_ (200 *μ*M) for 4 h and subsequently immunostained for activated Bax (red). Nuclei were counterstained by DAPI. (**c**) Cytosolic extracts were isolated from the transfected HeLa cells (as in **b**) after treatment with H_2_O_2_ (200 *μ*M) for 4 h and cytochrome c and caspase-9 were detected in the cytosol of cells by WB. (**d**) Early apoptosis was measured in transfected HeLa cells (as in **b**) after treatment with H_2_O_2_ (200 *μ*M) for 4 h by annexin V staining. (**e**) HeLa cells were transfected with specific siRNA targeting human CerS6 or scrambled control siRNA for 24 h (transfection efficiency was above 90%) and subjected to KCN (1 mM) for additional 24 h (left panel). 143B_ΔCOX_ cells were transfected with siRNA against human CerS6 or scrambled control siRNA (middle panel; transfection efficiency was above 80%). COX10^−/−^ cells were transfected with siRNA against murine CerS6 or scrambled control siRNA (right panel; transfection efficiency was about 30%). Cells were then exposed to H_2_O_2_ as indicated and cell death was measured by trypan-blue exclusion after 24 h. In order to obtain significant cytotoxic response (low transfection efficiency), COX10^−/−^ cells were treated with 300 *μ*M H_2_O_2_ as indicated. (**f**) HeLa and 143B_ΔCOX_ cells (as in **e**) were subjected to H_2_O_2_ for 6 h and cytochrome c was detected in cytosolic extracts by WB. The error bars represent the mean±S.D. (*n*=3), two-tailed unpaired *t*-test. *P<0.05, **P <0.01, ***P<0.001

Six mammalian ceramide synthases have been identified so far, each utilizing distinct fatty acyl CoA esters of more or less defined chain lengths for *N*-acylation of the sphingoid long-chain base.^29^ Therefore, we examined whether COX dysfunction impacts on transcriptional regulation of ceramide synthases. The qRT-PCR analysis revealed a specific transcriptional upregulation of CerS1, 3 and 6 upon COX dysfunction (Figure 4a). Among different ceramide species and their respective ceramide synthases, CerS6 and its specific product C_16 : 0_ ceramide have been repeatedly shown to be involved in Bax/Bak-mediated mitochondrial apoptosis.^21, 30, 31^ Similar to qRT-PCR, our western blot analysis confirmed a significant upregulation of CerS6 protein level in all COX-deficient cells (Supplementary Figure 4A). In order to examine the role of the elevated CerS6 in oxidative stress-induced mitochondrial apoptosis, HeLa cells were first transiently transfected with DNA constructs encoding either GFP-tagged CerS6, the enzymatic inactive mutant of CerS6_R131A_,^32^ CerS1 (generating C_18_-ceramide), CerS3 (generating C_24_ and C_26_-ceramides), or GFP alone. Transfected cells were subsequently subjected to H_2_O_2_ and mitochondrial apoptosis was analyzed microscopically by examining Bax conformational change/activation (Figure 4b), by western blot analysis of cytosolic cytochrome c and activation of caspase-9 (Figure 4c) and by annexin V staining (Figure 4d). These data showed that only the overexpression of wild type CerS6 provoked mitochondrial apoptosis when cells were exposed to H_2_O_2_. In contrast, the enzymatically inactive CerS6_R131A_ variant, CerS1 or CerS3 failed to induce Bax activation, the release of cytochrome c or activation of caspase-9. Notably, similar to CerS6, CerS5 is responsible for the production of C_16 : 0_ ceramide.^33, 34^ Accordingly, the overexpression of CerS5 efficiently provoked the release of cytochrome c, caspase-9 activation and apoptosis in HeLa cells (Figures 4c and d) underscoring the pivotal role of C_16 : 0_ ceramide in this process.

The specific role of CerS6 was further examined by using CerS6-specific siRNAs. Accordingly, specific knockdown of CerS6 completely abolished the increased susceptibility of KCN-treated HeLa, 143B_ΔCOX_ and COX10^−/−^ cell lines to H_2_O_2_ (Figure 4e and Supplementary Figure 4B). In line with these observations, H_2_O_2_-induced cytochrome c release was significantly reduced when CerS6 was downregulated in KCN-treated HeLa or 143B_ΔCOX_ cells (Figure 4f). Importantly, as a nontarget gene of COX dysfunction (Figure 4a), the specific knockdown of CerS5 did not alter the susceptibility of KCN-treated HeLa cells to H_2_O_2_ (Supplementary Figure 4C). These data clearly indicate that the accelerated CerS6 expression and its increased activity upon COX inhibition are responsible for the enhanced mitochondrial apoptosis in response to oxidative stress.

## Discussion

Since the first discovery of diseases-causing mutations in OXPHOS components,^35, 36^ extensive research efforts have focused to address their pathophysiology.^37^ Meanwhile, accelerated apoptotic cell death has been considered as one of the major causes of degenerative state upon mitochondrial dysfunction.^2^ However, whether the decline in mitochondrial respiratory activity in general or specific alterations of RC complexes directly interferes with cellular death decision remains largely unknown. Here we showed for the first time that the specific inhibition of complex IV results in an increased susceptibility to oxidative stress, indicating a unique mode of crosstalk between COX and the cell death machinery during oxidative damage. Previous data showed that mitochondrial diseases are not solely caused by a general depletion of ATP or dysfunctional OXPHOS, but rather by differentially interfering with cellular stress responses. Whereas deficiencies in respiratory complexes I, III, V or CoQ increased cellular ROS levels and induced oxidative damage, complex IV dysfunction did not promote ROS generation, and the cell death induced by COX dysfunction was rather due to the increased susceptibility of these cells to additional (environmental) stress cues.^17^ Accordingly, genomic ablation of *cox10* in liver resulted in COX deletion and severe OXPHOS defects but did not directly induce hepatocyte apoptosis. COX10-deficient hepatocytes, however, were markedly susceptible to exogenous stress involving death receptor (FAS)-induced apoptosis.^38^ Together these observations indicate the specific mode of cellular stress responses when complex IV is inhibited. Eventually, based on the apoptotic capability of a cell and its regenerative potential, COX dysfunction may result in an efficient elimination of damaged cells and in tissue renovation in some tissues (e.g. liver) ^38^ or accumulation of dysfunctional cells and progressive decline in tissue function (e.g. muscle).^39^ This may in turn impact on tissue segregation of mtDNA mutations disabling COX function.

**Figure 5:**
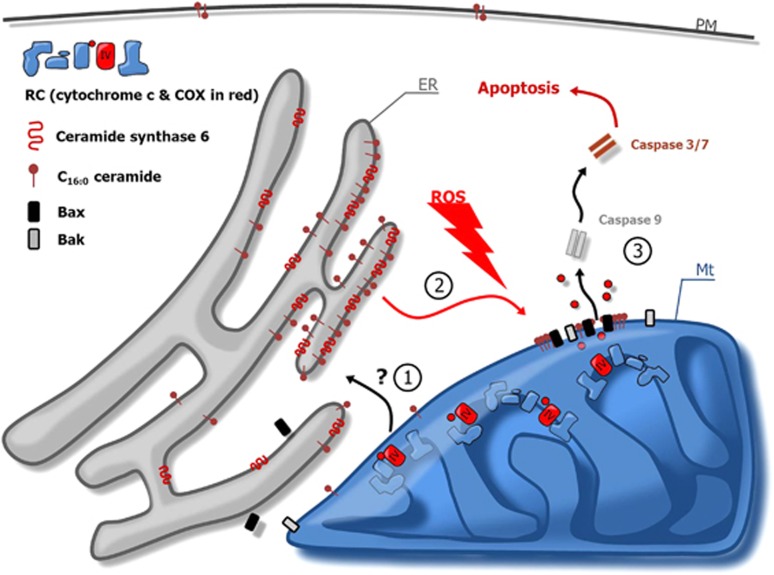
Proposed model of COX deficiency-induced apoptosis in response to oxidative stress. COX deficiency leads by an as yet unknown mechanism to enhanced expression of CerS6 (mainly associated with ER membranes) and accumulation of intracellular C_16 : 0_ ceramide (1). The increased intracellular C_16 : 0_ ceramide promotes the release of mitochondrial cytochrome c by involving Bax and Bak (2). The cytosolic cytochrome c induces the activation of initiator caspase-9 and downstream executioner caspases, finally resulting in apoptosis of the COX-deficient cell (3). Mt, mitochondria; PM, plasma membrane; RC, respiratory chain.

One of the most well-known theories explaining tissue degeneration and aging upon mitochondrial dysfunction is the free radical theory of aging, which was first proposed by Harman in 1956.^40, 41, 42^ Mitochondria are one of the major sources of ROS but in particular represent an important cellular target of oxidative stress.^43^ In addition to its cytotoxic effects, physiologic levels of ROS have a pivotal role in cellular signaling and homeostasis, which ultimately guarantee tissue function. Oxidative lethality occurs only when ROS levels increase to high levels or when cellular integrity and fitness are altered, for example, upon aging.^10^ In line with this notion our data showed that when COX was inhibited chemically or genetically, cellular susceptibility to ROS was markedly increased (Figure 1). The elevated susceptibility to ROS was mediated by Bax/Bak-dependent mitochondrial apoptosis (Figure 2), whereas no alteration in the expression levels of Bcl-2 family members was observed (Supplementary Figure 2). Previous data demonstrated that Bax/Bak-dependent release of cytochrome c from mitochondria can be efficiently facilitated by ceramides, in particular, the specific product of CerS6, C_16 : 0_ ceramide.^19, 20, 21, 22, 23, 30, 31^ Our data identified the transcriptional upregulation of CerS6 as a key cellular stress response to COX inhibition, which generated C_16 : 0_ ceramide, promoted cytochrome c release and increased the susceptibility to oxidative stress (Figures 3 and 4). However, the molecular path, which links COX to *cerS6* gene activation remained undetermined and requires further investigations. Importantly, these data do not exclude the possible involvement of nontranscriptional cellular regulatory circuits impacting on CerS6 enzymatic activity upon COX dysfunction. Independently, increased cellular ceramide levels specifically promotes permeability of mitochondrial outer membrane either by involving Bcl-2 proteins^19, 20, 21, 22, 23, 30, 31^ or alone by formation of large protein-permeable channels in mitochondrial membranes.^27^ Accordingly, the identification of ceramide synthase activity upon COX dysfunction provides a novel concept about how mitochondrial disorder interferes with the cellular death machinery and causes tissue degeneration (Figure 5).

The first CerS (CerS1) was initially described in yeast as longevity assurance gene 1 (LAG1), as its deletion prolonged yeast chronological lifespan.^44^ LAG1 homologue (LASS1) was then identified in human and mouse and was shown to be able to restore lifespan in a yeast strain lacking LAG1 and its cognate LAC1 (longevity assurance gene 1 cognate).^45^ Subsequently, LAG1 and LAC1 were shown to be necessary for yeast ceramide synthesis,^46, 47^ and extensive biochemical studies identified six mammalian CerSs (CerS1-6) displaying specificity toward fatty acyl CoAs of more or less defined chain length.^29^ Despite significant advances during the past decade concerning the involvement of ceramide synthesis in human diseases,^48^ the pathophysiologic role of CerSs in human aging has not yet been elucidated. Aging is characterized by a progressive decline in organismal functions. Mitochondrial dysfunction has been considered as one of the central causes of aging and aging-associated diseases. Here we explored a novel role of CerS6 in cellular death and tissue degeneration caused by mitochondrial dysfunction due to COX deficiency. A potential therapeutic benefit of manipulating the apoptotic machinery has been postulated in degenerative diseases including Alzheimer's Disease.^49, 50^ On the basis of its pivotal role in regulating cell death upon COX dysfunction, CerS6 might potentially represent a novel target for therapeutic intervention in mitochondrial diseases caused by COX dysfunction. In line with this notion, CerS6 was recently highlighted as a promising therapeutic target in metabolic disorders including glucose intolerance and insulin resistance.^51, 52^

## Materials and Methods

### Cell culture

The human osteosarcoma cybrid cell line 143B_ΔCOX_ contains a homoplasmic G to A transition at position 6930 of the mtDNA, leading to a loss of the last 170 amino acids of COX subunit I (COXI)^12^ and to rapid disassembly of COX-containing supercomplexes.^53^ The murine fibroblast cell line COX10^−/−^ was generated by the transfection of skin fibroblasts of adult mice containing the floxed exon 6 of the assembly factor COX10 with a pCre-Hygro plasmid expressing the P1 Cre recombinase, resulting in the disassembly of the COX holoenzyme and complete knockout of COX.^39^ Human cell lines HeLa (CCL-2, obtained from American Type Culture Collection (ATCC), Rockville, MD, USA), HeLa Bcl-2,^16^ 143B control and 143B_ΔCOX_ as well as adult murine fibroblasts of COX10 knockout mice and murine embryonic fibroblasts (MEFs) of Bax/Bak double-knockout mice (gift from A. Villunger, Innsbruck, Austria) were cultured in Dulbecco's modified Eagle's medium (Gibco (Life Technologies), Darmstadt, Germany), supplemented with 10% fetal calf serum (Gibco (Life Technologies)), 100 *μ*/ml streptomycin and 100 U/ml penicillin (Biochrom (Merck), Darmstadt, Germany). 143B_ΔCOX_, COX10 knockout fibroblasts and mammalian cell lines under conditions of chemical suppression of the mitochondrial RC were supplemented with 1 mM sodium pyruvate (Biochrom (Merck)) and 50 *μ*g/ml uridine (Sigma-Aldrich, Seelze, Germany) in order to maintain redox homeostasis and replace the defect in pyrimidine nucleotide synthesis.

### Chemicals and reagents

zLEHD-fmk, zIETD-fmk and zVAD-fmk were obtained from Enzo Life Sciences (Lörrach, Germany). Rotenone, 3-nitropropionic acid (3-NP), antimycin A, KCN, oligomycin, FB1 and H_2_O_2_ were purchased from Sigma-Aldrich.

### Measurement of oxygen consumption

Measurement of oxygen consumption was performed with a Clark-type oxygen electrode system (Hansatech Instruments, Norfolk, UK).^54^ In total, 4 × 10^6^ cells were suspended in air-saturated buffer E (300 mM mannitol; 5 mM MgCl_2_; 10 mM KCl; 10 mM KH_2_PO_4_, pH 7.4; and 1 mg/ml bovine serum albumin, fatty acid free) and placed into a water-jacketed chamber at 37 °C. Oxygen consumption under conditions of chemical inhibition of the respective RC complexes was recorded, related to total cell number and expressed as femtomoles O_2_ per cell per min.

### Transient overexpression and siRNA-mediated knockdown

For the construction of human ceramide synthase (CerS), GFP fusion proteins, open reading frames encoding human ceramide synthase 1, 3 and 6 and 6_R131A_ were amplified by PCR with appropriate restriction sites and cloned into pEGFP-N3 vector (Clontech, Saint-Germain-en-Laye, France). Cells were transfected with the TurboFect transfection reagent (Thermo Scientific, Rockford, IL, USA) according to the manufacturer's manual and incubated for at least 24 h. For siRNA-mediated knockdown of human CerS6 (5′-AAGGUCUUCACUGCAAUUACATT-3′)^55^ as well as murine CerS6 (5′-GAGGAGAAACCCAGCACUC-3′), cells were transfected with 100 pmol/ml siRNA using Lipofectamine RNAimax transfection reagent (Life Technologies) and incubated for 48 h before treatment. All siRNAs were purchased from the Eurofins MWG Operon (Ebersberg, Germany).

### Measurement of cell death

Cell death was measured by trypan-blue exclusion. In brief, ~0.75–1.5 × 10^5^ cells were seeded in 24-well chambers and incubated under desired conditions. Extensive kinetics and dose–response analyses of H_2_O_2_ were performed to identify the concentrations of H_2_O_2_ with similar cytotoxic activity toward different cell lines derived from different origins. The data included were mainly derived from analyses using 50 *μ*M H_2_O_2_ in HeLa cells and 150 *μ*M H_2_O_2_ in 143B_ΔCOX_ and COX10^−/−^ cells. Before counting, cells were detached with trypsin and diluted to appropriate working concentrations. The cell suspension was then diluted with trypan-blue in a ratio of 1 : 1 (v/v) and dead cells were counted using an automated cell counter (Countess, Life Technologies) according to the manufacturer's instructions.^56^

### Measurement of superoxide formation

For microscopic analysis of superoxide (O_2_^−^) formation, the MitoSox Red mitochondrial superoxide kit (Life Technologies) was used according to the manufacturer's instructions. In brief, 3 × 10^5^ cells were seeded on sterile glass bottom live cell dishes (Greiner Bio-One, Frickenhausen, Germany), incubated with 5 *μ*M MitoSox reagent working solution and washed three times with warm HBSS/Ca/Mg buffer (Life Technologies). Samples were analyzed with an Olympus Fluoview 1000 confocal microscope (Olympus, Hamburg, Germany).

### Sample preparation and western blotting (WB)

Whole cell lysates were prepared by incubating cell pellets in CHAPS lysis buffer (10 mM HEPES, pH 7.4; 150 mM NaCl; 1% CHAPS; inhibitor) on ice for 20 min and subsequent centrifugation at 14 000 × *g* for 20 min at 4 °C to recover the supernatant. To obtain cytosolic extracts, cells were incubated for 20 min in ice-cold HEP buffer (20 mM HEPES, pH 7.5; 10 mM KCl; 1.5 mM MgCl2; 1 mM EDTA; 10 *μ*M cytochalasin B; 1 mM DDT; protease inhibitor) following mechanical disruption by repeated passaging ( × 12–15) through a 26 × G 1/2̀̀ needle and the cytosolic supernatant was recovered after centrifugation at 20 000 × *g* for 20 min at 4 °C.^57, 58^ For WB, equal amounts of protein, determined by BCA protein assay (Thermo Scientific, Rockford, IL, USA) were prepared in 1 × Laemmli buffer containing 4% ß-mercaptoethanol, separated by SDS-PAGE and transferred onto nitrocellulose membranes (Protran BA85, GE Healthcare, Freiburg, Germany). After blocking and appropriate incubation with primary and secondary antibodies, protein signals were visualized by enhanced chemiluminescence (Thermo Scientific, Rockford, IL, USA). Mouse monoclonal anti-actin antibody was obtained from Sigma-Aldrich. Mouse monoclonal anti-Bak (clone G317-2), mouse monoclonal anti-active Bax (clone 6A7), rabbit polyclonal anti-Bax, mouse monoclonal anti-Bcl-2 (clone 4D7) and mouse monoclonal anti-cytochrome C (clone 7H8.2C12) were obtained from BD Bioscience (Heidelberg, Germany). Mouse monoclonal anti-Bcl-xL (clone 5H46), rabbit monoclonal anti-caspase-3 (clone 8G10), rabbit polyclonal anti-caspase-9 (No. 9502 human-specific and No. 9504 mouse-specific), rabbit monoclonal anti-GAPDH (clone D16H11) and rabbit polyclonal anti-Mcl-1 antibodies were obtained from Cell Signaling (Frankfurt a.M., Germany). mAb against COXI (clone 1D6E1A8) was obtained from Mitoscience/Abcam (Cambridge, UK). Mouse monoclonal anti-complex II (clone 2E3GC12FB2AE2) antibody was obtained from Invitrogen (Karlsruhe, Germany). Rabbit polyclonal anti-CerS6 was obtained from Abnova (clone A01; Taipei City, Taiwan).

### Immunoprecipitation of active Bax

100 *μ*g of whole cell lysates were brought to a final volume of 500 *μ*l with CHAPS lysis buffer containing 150 mM KCl and incubated with 2 *μ*g of monoclonal, active Bax-specific antibody 6A7 (BD Bioscience) overnight at 4 °C. The antigen–antibody complex was immobilized by the addition of GammaBind-G-Sepharose (GE Healthcare, Freiburg, Germany) and subsequent gentle rotation for 2 h at 4 °C. Samples were washed twice with CHAPS/150 mM KCl and the complexes were pelleted by centrifugation at 500 × *g* for 1 min. The resolved pellet was washed twice with CHAPS/150 mM KCl and further subjected to SDS-PAGE and WB.^58^

### Sphingolipid analysis

Total cellular ceramide and sphingomyelin contents were determined by liquid chromatography coupled to electrospray ionization tandem mass spectrometry (LC–ESI–MS/MS). Approximately 3 × 10^6^ cells were homogenized in 300 *μ*l of water using the Precellys 24 Homogenisator (Peqlab, Erlangen, Germany). Lipid extraction and LC–ESI–MS/MS analysis were performed as previously described.^59^ Endogenous ceramides and sphingomyelins with d18 : 1 sphingosine base were monitored in the positive ion mode with their specific multiple reaction monitoring (MRM) transitions ([M+H]^+^→*m/z* 264 for ceramides and [M+H]^+^→*m/z* 184 for sphingomyelins).^60^ Ceramide and sphingomyelin species were quantified on the basis of calibration curves, which were calculated from LC–MS/MS measurements of serially diluted synthetic ceramide and sphingomyelins standards (Avanti Polar Lipids, Alabaster, AL, USA). Linearity and correlation coefficients of the standard curves were obtained via linear regression analysis. The standard curves were linear in a range between 0.0 and 100 pmol on column with correlation coefficients (*R*^2^)>0.98. The calculated amounts of endogenous sphingolipids were normalized to the protein content of the cell homogenate.

### Ceramide synthase and sphingomyelinase activity measurement

A total of 2.5 × 10^6^ cells was incubated with 2 *μ*M d17 : 0 dihydrosphingosine (Avanti Polar Lipids, Alabaster, AL, USA), which is a not naturally occurring sphingoid base, for 4 h. Cell homogenization, lipid extraction and LC–ESI–MS/MS measurement were performed as described above. Ceramides with d17 : 1 sphingosine base were monitored in the positive ion mode with their specific MRM transitions ([M+H]^+^→*m/z* 250). As ceramides with d17 : 1 sphingosine base are not commercially available, those ceramide species were quantified on the basis of calibration lines calculated from LC–MS/MS measurements of synthetic d18 : 1 ceramide standards (see above).

The activity of neutral and acid sphingomyelinase (n/aSMase) was measured from crude cytosolic extracts (nSMase) or total cell lysates (aSMase) of at least 3 × 10^6^ cells in appropriate buffer using 0.2 *μ*Ci/ml [^14^C]sphingomyelin (Amersham/GE Healthcare).^21^ The amount of [^14^C]phosphorylcholin derived from sphingolipid hydrolysis was measured by thin layer chromatography and scintillation counting (Zinsser Analytic, Frankfurt a.M., Germany).

### Fluorescence microscopy

For immunofluorescence analyses of Bax activation and cytochrome c release, 3 × 10^5^ HeLa cells were transfected with 1 *μ*g of the particular pEGFP-N3 constructs and incubated for 24 h before subjection to H_2_O_2_ for 4 h.^58^ Cells were washed twice with PBS, fixed in 3% paraformaldehyde and simultaneously permeabilized and blocked with blocking buffer (3% BSA, 0.1% saponin in PBS) for 30 min. Cells were incubated with monoclonal active Bax 6A7 antibody (BD Bioscience) overnight at 4 °C. Cells were washed three times for 10 min with washing buffer, incubated with Alexa Fluor 647 secondary antibody (Life Techonlogies) for 1 h at room temperature and washed three times with washing buffer for 10 min. DAPI was added to the second washing step for nuclei staining. Samples were mounted onto cover slides using Mowiol mounting medium and examined with a spinning disk confocal microscope (Perkin Elmer, Rodgau, Germany).^61^

### qRT-PCR

Total RNA from COX10^fl/fl^ (control) or COX10^−/−^ fibroblasts was isolated using the standard phenol/chloroform method.^56^ cDNA was synthesized by cDNA First-Strand Aid Kit (Fermentas, Thermo Fisher Scientific, Waltham, MA, USA) using Oligo(dT)primers according to the manufacturer's instructions. qRT-PCR was performed with primers specific for murine GAPDH (forward 5′-TCA CCA CCA TGG AGA AGG C-3′ reverse 5′-GCT AAG CAG TTG GTG GTG CA-3′), CerS1 (forward 5′-GCC ACC ACA CAC ATC TTT CGG-3′ reverse 5′-GGA GAC GGT AAG CGC AGT AG-3′), CerS2 (forward 5′-AGA GTG GGC TCT CTG GAC G-3′ reverse 5′-CCA GGG TTT ATC CAC AGT GAC-3′), CerS3 (forward 5′-CCT GGC TGC TAT TAG TCT GAT G-3′ reverse 5′-CTG CTT CCA TCC AGC ATA GG-3′), CerS4 (forward 5′-CTG TGG TAC TGT TGT TGC ATG AC-3′ reverse 5′-GCG CGT GTA GAA GAA GAC TAA G-3′), CerS5 (forward 5′-TGG CCA ATT ATG CCA GAC GTG AG-3′ reverse 5′-GGT AGG GCC CAA TAA TCT CCC AGC-3′) and CerS6 (forward 5′-GCA TTC AAC GCT GGT TTC GAC-3′ reverse 5′-TTC AAG AAC AGG ACT CCG TAG-3′). qPCR was performed using SYBR Green Mix (Roche Applied Sciences, Penzberg, Germany) using a 96-well-plate multicolor real-time PCR detection system (CFX96 Touch, BioRad). Data from three experimental replicates were evaluated using the Pfaffl method.

## Abbreviations

RC: respiratory chain
COX: cytochrome c oxidase
CerS6: ceramide synthase 6
OXPHOS: oxidative phosphorylation
IMM: inner mitochondrial membrane
mtDNA: mitochondrial DNA
nDNA: nuclear DNA
ROS: reactive oxygen species
SMase: sphingomyelinase
FB1: fumonisin B1
LAG1: longevity assurance gene 1
LAC1: longevity assurance gene 1 cognate
