# Supplementary material for: Cytochrome c oxidase deficiency accelerates mitochondrial apoptosis by activating ceramide synthase 6
Source: Cell Death Dis. 2015 Mar 12;6(3):e1691–. doi: 10.1038/cddis.2015.62 (PMC4385940; doi:10.1038/cddis.2015.62)
Supplement: Supplementary Information [file cddis201562x1.pdf]

SUPPLEMENTARY INFORMATION

Figure S1: HeLa cells were subjected to increasing concentrations of H_2O_2 and cell death was assayed by trypan-blue exclusion after 24h. Error bars represent the mean ± SD (n=3).

Figure S2: (A) COX-deficiency does not enhance superoxide formation. HeLa cells were incubated with KCN [1mM], rotenone [25nM] or 2% (v/v) DMSO for 2h. 143B cybrids cells and COX10 fibroblasts were incubated with 25 nM rotenone (rot) for 3h. Superoxide formation was detected by MitoSOX and visualized by confocal microscopy. (B) HeLa cells were pretreated with KCN [1mM] for 48h or left untreated. Western blot analysis of indicated Bcl-2 protein family members in HeLa and 143B cybrid cell lines.

Figure S3: (A) Total sphingomyelin content in COX-deficient cells and their respective controls was analyzed by mass spectrometry of total cell homogenates. HeLa cells were pretreated with KCN [1mM] for 48h or left untreated. (B) Neutral and acid SMase activity was measured from crude cytosolic extracts (nSMase) or total cell lysates (aSMase). HeLa cells were pretreated with KCN [1mM] for 48h or left untreated. (C) COX-deficient $143B_{\Delta COX}$ cells were incubated in the presence of the ceramide synthase inhibitor FB1 for 48h. Total ceramide and sphingomyelin contents were analyzed by mass spectrometry of total cell homogenates. Error bars represent mean ± SD of 2 analytical replicates of 3 biological replicates (HeLa and 143B) and mean ± SD of 2 analytical replicates of 2 biological replicates (COX10), respectively. **p<0.01, ***p<0.001

Figure S4: (A) Western blot analyses of CerS6 in COX-deficient cells and their respective controls. HeLa cells were pretreated with KCN [1mM] for 48h. (B) Efficiency of knockdown of CerS6 by increasing concentrations [25nM; 50nM; 75nM] of siCerS6 was measured in HeLa cells after 72h by Western blotting (left panel). Efficiency of knockdown of CerS6 by increasing concentrations [25nM; 50nM; 75nM] of siCerS6 was measured in 143B control cells after 72h (middel panel). Efficiency of knockdown of CerS6 by increasing concentrations [50nM; 75nM] of siCerS6 was measured in $COX10^{FL/FL}$ cells after 48h (right panel). (C) HeLa cells were transfected with specific siRNA targeting human CerS5 or scrambled control siRNA for 24h and subjected to KCN [1mM] for additional 24h. Cells were subjected to H_2O_2 and cell death was measured by trypan-blue exclusion after 20h. The error bars represent the mean ± SD (n=3), two-tailed unpaired *t*-test. *p <0.05, ***p<0.001

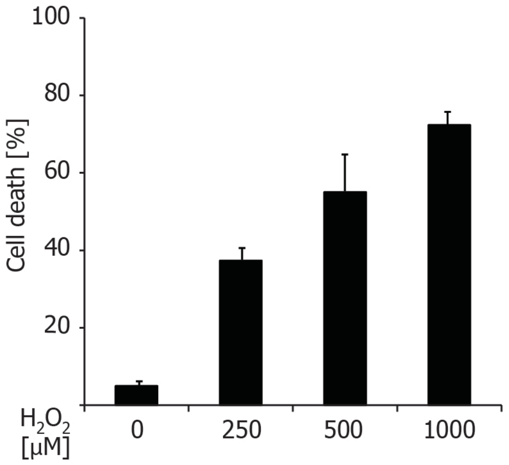

Figure S1

A

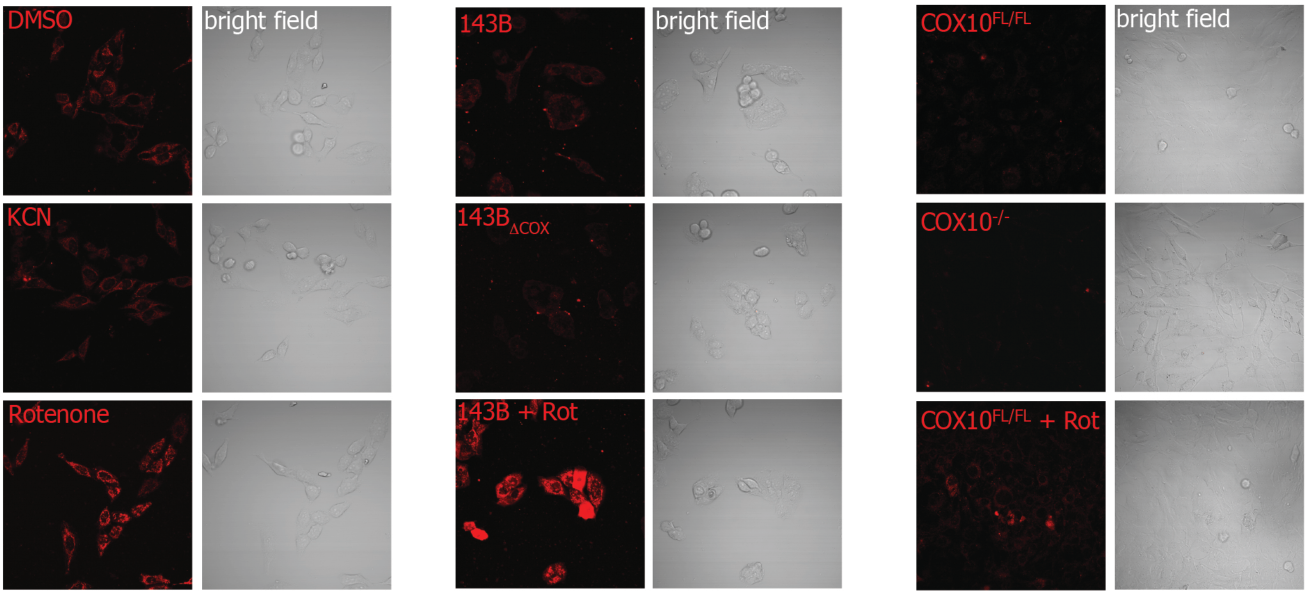
DMSO
bright field
KCN
Rotenone
143B
bright field
143B$_{\Delta COX}$
143B + Rot
COX10$^{FL/FL}$
bright field
COX10$^{-/-}$
COX10$^{FL/FL}$ + Rot

B

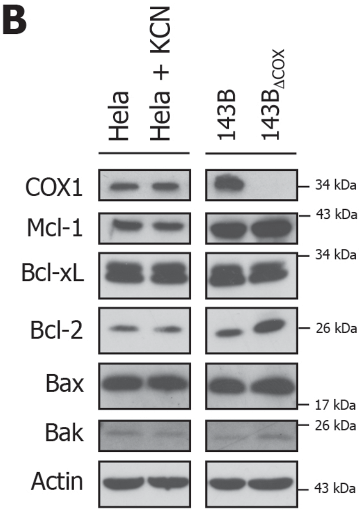
Hela
Hela + KCN
143B
143B$_{\Delta COX}$
COX1
Mcl-1
Bcl-xL
Bcl-2
Bax
Bak
Actin
34 kDa
43 kDa
34 kDa
26 kDa
17 kDa
26 kDa
43 kDa

Figure S2

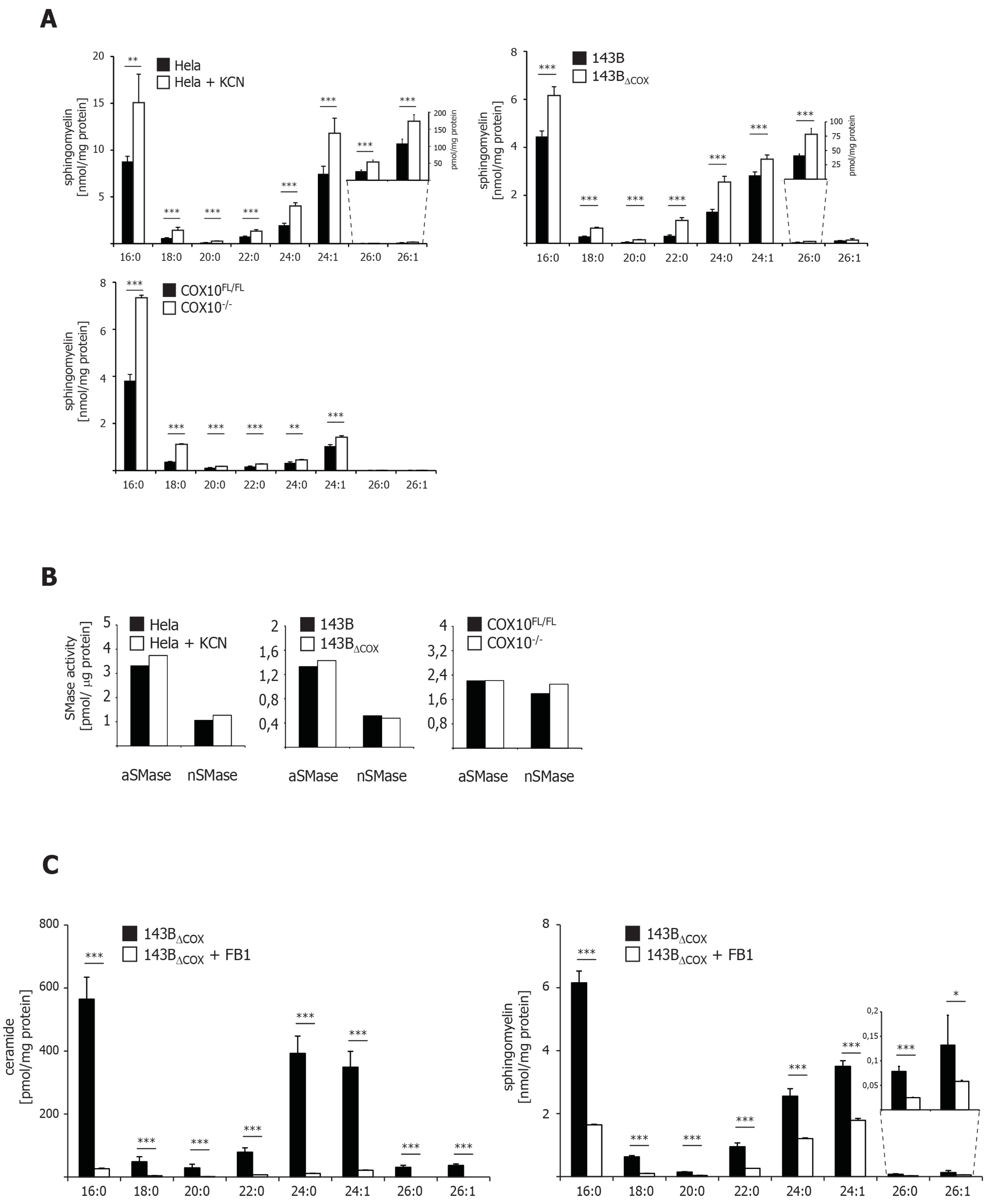
A
Hela
Hela + KCN
sphingomyelin [nmol/mg protein]
pmol/mg protein
143B
143BΔCOX
COX10FL/FL
COX10-/-
16:0
18:0
20:0
22:0
24:0
24:1
26:0
26:1
B
SMase activity [pmol/ µg protein]
aSMase
nSMase
C
143BΔCOX
143BΔCOX + FB1
ceramide [pmol/mg protein]
sphingomyelin [nmol/mg protein]

Figure S3

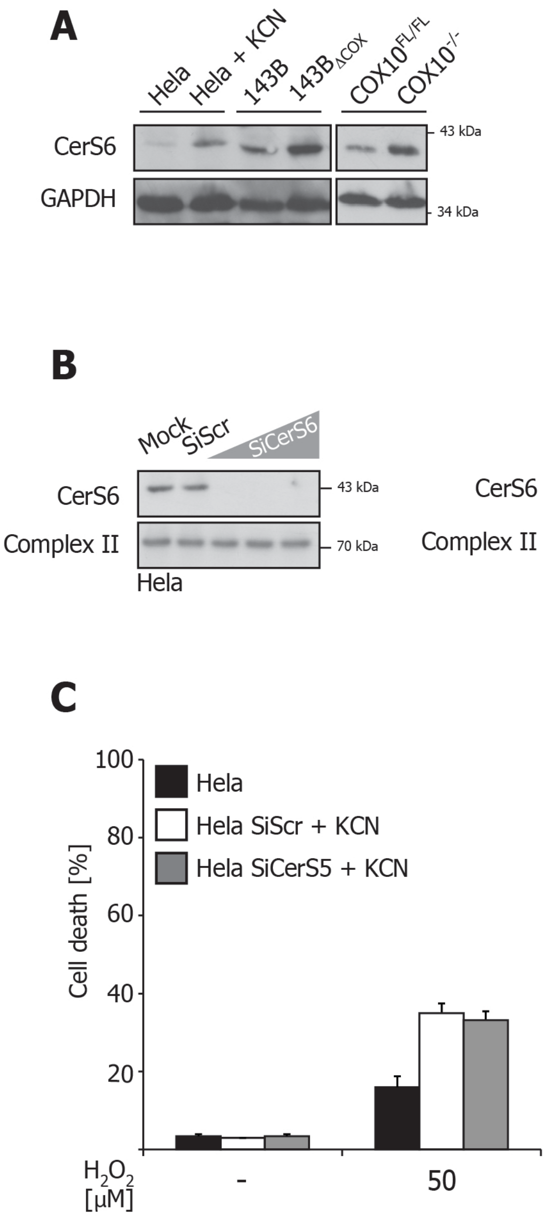
A
Hela
Hela + KCN
143B
143B ΔCOX
COX10 FL/FL
COX10 -/-
CerS6
GAPDH
43 kDa
34 kDa
B
Mock
SiScr
SiCerS6
CerS6
Complex II
43 kDa
70 kDa
Hela
143B
GAPDH
34 kDa
COX10 FL/FL
C
Hela
Hela SiScr + KCN
Hela SiCerS5 + KCN
Cell death [%]
100
80
60
40
20
H2O2 [µM]
-
50

Figure S4
